# Incidence of PTSD in the French population a month after the COVID-19 pandemic-related lockdown: evidence from a national longitudinal survey

**DOI:** 10.1186/s12889-022-13880-9

**Published:** 2022-08-05

**Authors:** Caroline Alleaume, Patrick Peretti-Watel, François Beck, Damien Leger, Guillaume Vaiva, Pierre Verger, the COCONEL Group, Patrick Peretti-Watel, Valérie Seror, Sébastien Cortaredona, Odile Launay, Jocelyn Raude, Pierre Verger, Caroline Alleaume, Lisa Fressard, Guillaume Vaiva, François Beck, Stéphane Legleye, Damien Léger, Olivier L’Haridon, Jeremy K. Ward

**Affiliations:** 1 Southeastern Health Regional Observatory (ORS Paca), Faculté des Sciences Médicales et Paramédicales, 27 Bd Jean Moulin 13385, CEDEX 5 Marseille, France; 2 Aix-Marseille University, IRD, AP-HM, SSA, VITROME, Marseille, France; 3 CESP, University Paris Sud, Faculté de médecine UVSQ, Inserm, University Paris-Saclay, Villejuif, France; 4 Université de Paris, EA 7330 VIFASOM (Vigilance Fatigue Sommeil et Santé Publique), Paris, France; 5 APHP- Hôtel-Dieu, Centre du sommeil et de la Vigilance, Paris, France; 6 U1172 INSERM Lille Neurosciences & Cognitions, Centre National de Ressources & Résilience pour les psychotraumatismes (Lille – Paris), Lille, France

**Keywords:** Covid-19, PTSD, Lockdown, PCL-5, COCONEL cohort surveys

## Abstract

**Background:**

In view of experts’ warnings about the potential negative mental health consequences of the sudden nationwide lockdowns implemented in many countries to limit the spread of the COVID-19 pandemic, we sought to study the incidence of posttraumatic stress disorder (PTSD) after traumatic events related to this unprecedented lockdown in the French general population.

**Methods:**

This longitudinal study among adults (aged =18) consisted of two surveys: the first during the last days of the lockdown and the second a month later. We estimated PTSD incidence with the PCL-5 and ran multiple Poisson regression models to identify factors associated with PTSD.

**Results:**

Among the 1736 participants, 30.1% reported at least one traumatic event. PTSD incidence was 17.5% (95% confidence interval CI = 15.7–19.3). It was higher in participants who reported multiple traumatic events, who had high COVID-19-related media use, who had general anxiety disorder (GAD-7) during the lockdown, and who had GAD, depression (PHQ-9), or sleep problems 1 month later. In addition, 43.1% of people with PTSD reported suicidal thoughts.

**Conclusions:**

These results should help clinicians to target people who are at high risk of developing PTSD after a pandemic-related lockdown and could benefit from preventive measures. Collaboration between the media and mental health professionals could be envisioned to inform the population about care resources. Follow-up recommendations should also be disseminated to general practitioners to facilitate PTSD screening and ensure that they are aware of the appropriate management.

**Supplementary Information:**

The online version contains supplementary material available at 10.1186/s12889-022-13880-9.

## Introduction

The Coronavirus disease-2019 (COVID-19) emerged in late 2019 in China and spread rapidly in early 2020, and continued to circulate actively for the next 2 years, affecting million people worldwide with more than 535 million confirmed cases and leading to 6 million deaths by June 2022 [1]. With France among the European countries most strongly affected by this disease in 2020, French health authorities, like those of many countries, decided in March 2020 to implement a generalized nationwide lockdown. It started on March 17 and ended on May 10. Experts warned early on about the risks of increased psychological disorders, including posttraumatic stress disorders (PTSD) [2, 3]. Previous studies from China showed that PTSD was one of the most prevalent long-term psychiatric disorders among severe acute respiratory syndrome (SARS) survivors [4]. In view of the unprecedented magnitude of the COVID-19 pandemic, even as early as April, authors recommended investigation of PTSD symptoms in the general population and among health professionals [3]. Since, literature has tended to confirm experts’ fears, with variations by country, epidemic intensity and specific population groups [5–13]. A month after the lockdown began, PTSD prevalence was 2.7% among home-isolated Chinese university students [6] and 6.1% among the Chinese population aged 17-63, with youth, women, and people with responsibilities and concern for others more vulnerable to these symptoms [8]. PTSD estimates in Europe include 28% among adult Italian COVID-19 survivors 1 month after hospital discharge [9], 27.5% in the Italian general population during the first month of lockdown [10], and 15.8% in the Spanish general population during the equivalent period there [11]. In France, the prevalence of probable PTSD was found around 19.5% among university students 1 month after the COVID-19 lockdown [12], and 21.2% among hospital workers at least 3 months after the lockdown [13]. To our knowledge, no studies have been published yet on the PTSD incidence in the French general population in relation to COVID-19, although recent studies have shown an increase in the prevalence of COVID-19-associated psychological disorders (anxiety and depression) and sleep difficulties [14, 15].

To expand our knowledge of COVID-19’s consequences on mental health in France, we conducted the COCONEL cohort survey to 1) quantify the incidence of PTSD in the general population, 2) characterize the relative frequency of the PTSD symptoms observed, 3) study the sociodemographic, economic, and COVID-19-related factors, including media use to obtain information about the pandemic, associated with PTSD onset, and 4) study PTSD’s comorbidity with generalized anxiety and depression.

## Method

### Design and sample

**Fig. 1:**
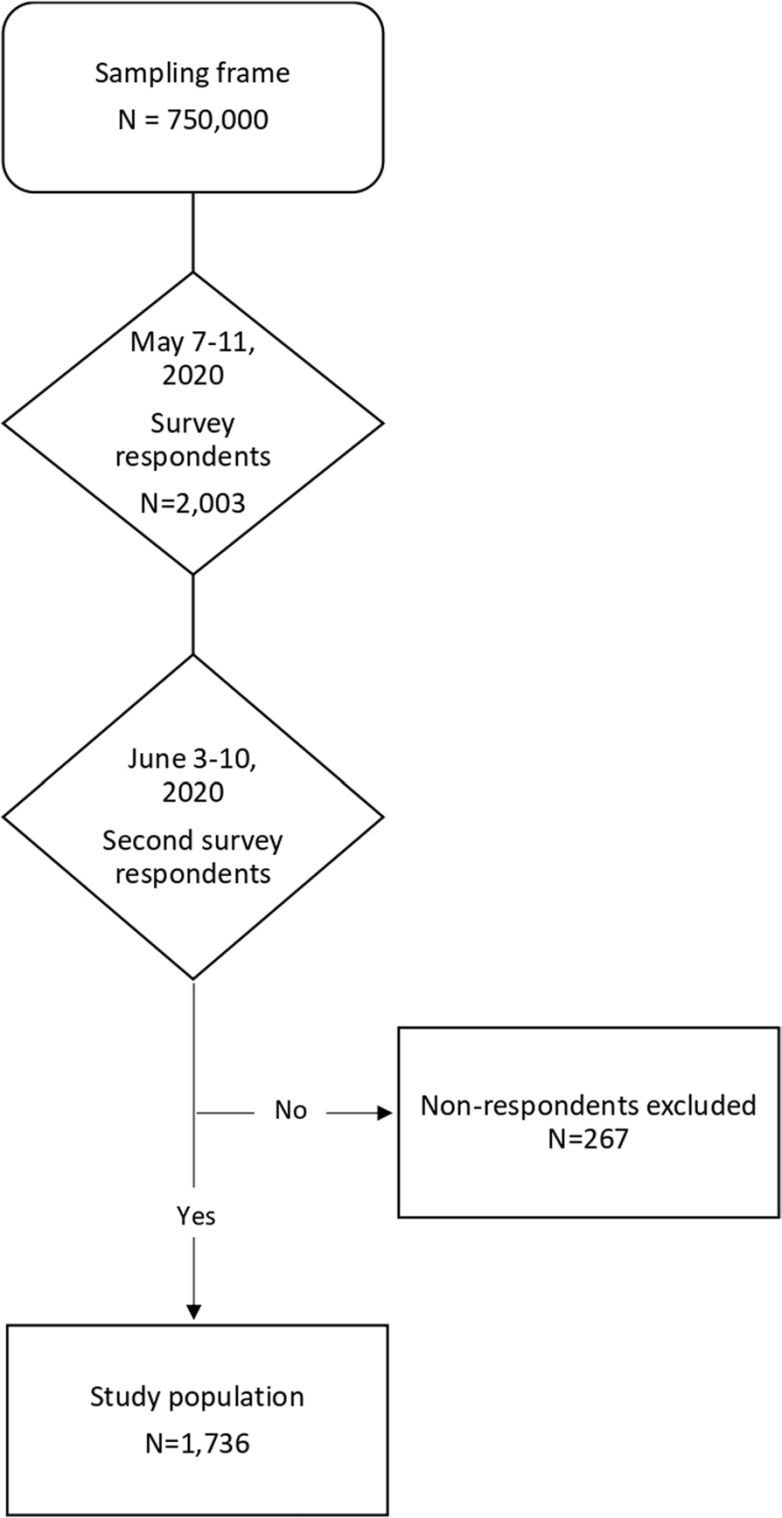
Flowchart for the enrolment and follow-up of subjects in this study^1^. ^1^The first sample (Mat 7-11, 2020 survey respondents) has been constituted using the quota method

The COCONEL cohort survey used two successive surveys, one taken during the last days of the first French lockdown (May 7-11, 2020) and the other about a month later (June 3-10, 2020) among the same representative sample of adults (18+ years old) residing in mainland France. The sample was selected using the quota method from an online research panel of more than 750,000 households, developed and maintained by IFOP (Polling Institute, Paris, France). A quota sampling method was applied to obtain a sample representative of the adult general population in France for age, gender, occupation, and rural/urban residence. To limit selection bias, panelists with low response rates (i.e., aged between 18 and 24 years old, blue-collar workers, and intermediate occupations) were oversampled relative to the others. Finally, 2003 panelists participated in the first survey and, among them, 1736 (86.7%) responded to the second (see Fig. 1).

### Data collected

#### PTSD instrument

We identified PTSD with the PTSD Check List Scale for DSM-5 (PCL-5), a 20-item self-report instrument, validated in French [16] and corresponding to DSM-5 symptom criteria for PTSD [17]. Because the first PTSD criterion requires exposure to a traumatic (very stressful) situation at least 1 month before the occurrence of symptoms, we used the PCL-5 during the second survey only. The PCL-5 includes four subscales describing the clusters in the DSM-5 corresponding to reexperiencing, avoidance, negative cognition and mood, and arousal. Responses to each item are collected with Likert scales ranging from 0 (not at all) to 4 (extremely), with a total score ranging from 0 to 80. High scores indicate high PTSD levels. We measured PTSD with the following previously published approach [17]: score = 2 on at least one item of the reexperiencing subscale, one of the avoidance subscale, two of the negative cognition and mood subscale, and two of the arousal subscale. A cutoff score of 33 was also applied to measure PTSD incidence [17] to ensure comparability with other studies.

#### Exposure to a traumatic event during the lockdown

Experience of a traumatic event during the lockdown was documented during both surveys: respondents were asked whether they had faced such an event (yes/no); those who answered yes were asked if the event was associated with some of the government announcements, their work situation, a relative infected by the coronavirus, travel, conflict with a member of the household, and/or the death of a family member not due to Covid-19 (several answers possible). The repetition of these questions in the second survey allowed construction of a variable measuring the dynamics of the perception of the traumatic event during the lockdown as follows: we defined “acute stress” as reporting a traumatic event during the lockdown in the first survey only; and “persistent stress” as reporting a traumatic event in the second survey, whether participants had or had not reported an event during the first survey.

#### Other measures and collected information

In addition, we used the Patient Health Questionnaire-9 (PHQ-9, 9 items) and the General Anxiety Disorder-7 (GAD-7, 7 items) to screen for prevalent depression or generalized anxiety disorder (GAD) during the lockdown (first survey) and during the 2 weeks before the second survey [18, 19]. We used a cutoff point of 5 on the PHQ-9 to identify individuals with probable at least mild depression [18], and a cutoff point of 5 on the GAD-7 to identify probable at least mild GAD [19]. The last PHQ-9 item, about to suicidal thoughts, was also analyzed separately to study the association between PTSD and suicidal thoughts, which is frequently highlighted in the literature [20]. We assessed sleep disorders at follow-up with the following item: “Have you had sleep problems during the last 8 days: not at all/yes, a few/yes, a lot” [14, 21].

During the first survey, participants were also asked how much they worried about becoming infected (score from 0, not worried at all, to 10, very worried) and, in the second survey, if they had been diagnosed with COVID-19, and if any relatives had been admitted to an intensive care unit due to the disease. The first survey also addressed participants media consumption for information about COVID-19 during the lockdown, asking them how many hours per day (less than 30 minutes, 30 minutes to 1 hour, 1-2 hours, 2-3, 3-4, 4-5, > 5 hours) they had spent looking at information about it from five different media sources (television, radio, newspaper, online websites, and social media) in the past week; we constructed a media exposure indicator according to a previously published method [15]. As answers to these five items were positively correlated (Cronbach’s alpha: 0.80) we summed them to obtain a score, and we used its fourth quartile as an indicator of high media.

Finally, we collected participants’ gender, age, region of residence, education level, financial (level of income measured as equivalized household income (EHI) quartiles) and work situations before and during the lockdown, household composition, overcrowded housing (defined as a living area <  18 square meters per person or < 25 sq.m. for a single person), and the lockdown’s perceived impact on the household’s financial situation.

### Data analysis

We weighted data so that the structure of the weighted sample matched that of the French population for age, gender, occupation, and population density in the region of residence. All analyses were performed with these weights. Using Student t-tests and proportion comparison tests, we first calculated PTSD scores (global and by cluster) and estimated PTSD incidence by type of traumatic event experienced. We then compared PTSD incidence according to individual characteristics among two different groups: the entire sample, and those who reported a traumatic event in either survey (prerequisite for measuring PTSD). We first used Chi-2 tests to explore associations between individual characteristics and PTSD incidence and then tested them in multivariate analyses. Variables significant at *p* < 0.20 in the univariate analyses were eligible for the multivariate models. To obtain relative risks, we performed two multiple modified Poisson regression models: 1/ in the entire population, and 2/ only among those with a traumatic event, to estimate the PTSD risks associated with the type of traumatic event and the persistence of stress between the two surveys. We checked the risk of multicollinearity and the potential endogeneity bias with a Heckman selection model. Finally, only significant factors are shown, except for gender, which we used as a basic adjustment variable. All statistical analyses were performed with SAS version 9.4 (SAS Institute, Cary, NC).

## Result

### PTSD incidence

**Fig. 2:**
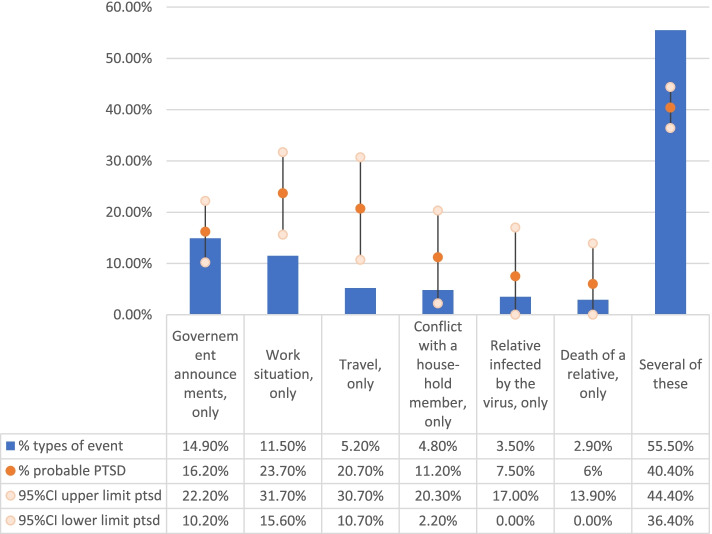
Types of stressful traumatic events reported by participants during the lockdown and PTSD incidence in each group (COCONEL, *N* = 1046). Population: Respondents to the May and June COCONEL surveys who reported a traumatic event during the lockdown (*N* = 1046). Note: Among participants who reported a traumatic event, 14.9% related it to governmental announcements only. Among the latter, the incidence of PTSD was 16.2%. The sum of the categories displayed is 98.3%; the missing 1.7% are people who selected open-ended responses that were too heterogeneous for analysis. Among those reporting several types of events, 72.3% selected at least government announcements, 62.3% their work situation, 52.7% travel, 47.7% conflict with a household member, 32.6% a relative infected with COVID-19, and 27.9% at least the death of a relative not due to COVID-19

During the first and/or second surveys, 1046 participants reported a traumatic experience during the lockdown: 34.5% only during the first survey, 5.5% during the second, 18.1% during both, 41.9% none, see Additional file 1: Figure 1). Most respondents reported several types of events (55.5%), 14.9% only events associated with some government announcements, and 11.5% only with their work situation (Fig. 2). Among people who reported several types of events, 72.3% included government announcements.

Among these 1046 participants, 30.1% were classified with PTSD 1 month after the lockdown ended. In the general adult population (Table 2) PTSD in incidence was 17.5% (95% CI = 15.7–19.3) with significantly higher rates among people reporting several types of events (*p* < 0.001, Fig. 1). The latter group was also more likely to meet each of the symptom subgroup criteria (Table 1). The cutoff score of 33 used in other publications [19] yielded a lower PTSD incidence estimate: 14.3% (95% CI = 12.7–16.0).

**Table 1:** Incidence of PTSD and of PTSD Symptom Clusters by Type of Traumatic Event Experienced during Lockdown (PCL-5, COCONEL 2020, *N* = 1046)^a,b^

	All types of events (*n*=1046)	Several events (*n* = 581)^c^	Government announce-ments (*n* = 156)		Work situation (*n* = 120)		Travel (*n* = 55)		Other (*n* = 134)	
	%	%	%	p	%	p	%	p	%	p
**PTSD**	30.1	40.4	16.2	< 0.001	23.7	< 0.001	20.7	< 0.001	11.2	< 0.001
Reexperiencing symptom group	49.2	58.7	43.6	< 0.001	41.9	< 0.001	32.0	< 0.001	28.3	< 0.001
Avoidance symptom group	41.7	52.8	29.5	< 0.001	33.4	< 0.001	29.2	< 0.001	20.4	< 0.001
Negative cognition and mood symptom group	47.6	57.7	35.1	< 0.001	43.3	0.004	33.5	< 0.001	27.9	< 0.001
Arousal symptom group	47.4	57.2	35.8	< 0.001	38.9	< 0.001	34.5	< 0.001	31.6	< 0.001

^a^Population: Respondents to the COCONEL May and June surveys who reported a traumatic event during the lockdown (*N* = 1046)

^b^Government announcements group, work situation group and travel group were separately compared to the multiple-events group

^c^Among people who reported a traumatic event related to government announcements, 43.6% met the reexperiencing group criteria and 16.2% the criteria for PTSD; these rates were lower than among people who reported several traumatic events (40.4% of them met full PTSD criteria, *p* < 0.001). The “Other” group included people reporting traumatic events related to a relative infected by COVID-19, or to a conflict with a household member, or to the death of a relative not related to COVID-19

### Factors associated with incidence of PTSD: univariate analyses

Incidence of PTSD did not differ according to gender (Table 2) but it was significantly higher in young people, those with low EHI, those confined with people other than their partner, and those who had never worked, both in the entire sample and among those exposed to traumatic events. PTSD incidence was higher in people reporting chronic health problems or a history of consultation for psychological disorders than among those who did not.

**Table 2:** Incidence of PTSD According to Sociodemographic Characteristics, Medical and Psychological History, Types of Stressors, and Psychiatric Comorbidity (COCONEL, *N* = 1736)^a^

Characteristics of the respondents	Incidence of PTSD			
	Among people exposed to a traumatic event (*n* = 1046)		Among the entire sample (*n* = 1736)	
	%	p	%	p
All (%)	30.1	–	17.5	–
**Sociodemographic characteristics**				
Gender		0.78		0.13
Men (*n* = 826)	30.5		16.0	
Women (*n* = 910)	29.7		18.8	
Age		0.008		< 0.001
< 35 years (*n* = 446)	37.0		23.2	
35–64 years (*n* = 863)	28.2		16.3	
> 64 years (*n* = 427)	25.7		13.9	
Education Level		0.02		0.14
No high school diploma (*n* = 880)	34.1		18.7	
High school diploma (*n* = 312)	25.7		14.7	
Undergraduate degree (*n* = 244)	30.9		20.1	
Postgraduate degree (*n* = 300)	23.1		14.5	
EHI		< 0.001		< 0.001
Low (q1) (*n* = 373)	41.5		23.9	
Middle (q2-q3) (*n* = 823)	28.5		16.6	
High (q4) (*n* = 371)	23.0		12.9	
Missing (*n* = 169)	27.9		17.5	
Financial difficulties due to the lockdown		< 0.001		< 0.001
Yes (*n* = 365)	45.4		32.5	
No (*n* = 1371)	24.7		13.5	
Confined in an overcrowded dwelling		< 0.001		< 0.001
Yes (*n* = 134)	45.5		31.8	
No (*n* = 1602)	28.5		16.3	
Condition of lockdown		< 0.001		< 0.001
Alone (*n* = 375)	28.5		15.6	
With one’s partner (*n* = 1102)	26.9		15.5	
With people other than one’s partner (*n* = 259)	44.0		28.7	
Work situation before the lockdown^b^	ns	0.49	#	0.07
Work (*n* = 909)	30.7		18.8	
Unemployed (*n* = 97)	32.1		20.8	
Retired (*n* = 506)	26.2		13.3	
Student (*n* = 102)	31.8		20.1	
Other inactive (*n* = 122)	36.1		19.9	
**Health conditions reported during the lockdown**				
Chronic health problem or chronic disease		< 0.001		< 0.001
Yes (*n* = 502)	37.5		23.5	
No, don’t know (*n* = 1234)	26.7		15.0	
Consulted for psychological issues in the 12 months before lockdown		< 0.001		< 0.001
Yes (*n* = 191)	48.2		41.1	
No (*n* = 1545)	26.6		14.6	
**Covid-19 exposure**				
Living in an area strongly impacted by Covid-19		0.04		0.007
Yes (*n* = 706)	33.4		20.4	
No (*n* = 1030)	27.6		15.5	
Diagnosed with Covid-19		< 0.001		< 0.001
Yes (*n* = 44)	62.0		50.1	
No (*n* = 1692)	28.9		16.6	
Relative admitted to an intensive care unit due to Covid-19		0.79	**	0.005
Yes	31.2		27.0	
No	29.9		16.8	
Media consumption per day of information about COVID-19 during the lockdown		< 0.001		< 0.001
Low (< 1 hr) (*n* = 415)	17.5		8.5	
Intermediate (1- < 4 hrs) (*n* = 871)	22.4		12.8	
High (= 4 hrs) (*n* = 450)	50.1		34.9	
Serious concern about being infected by COVID-19		< 0.001		< 0.001
Yes (*n* = 179)	42.8		29.5	
No (*n* = 1557)	26.9		15.0	
**Mental health symptoms**				
Anxiety (mild to severe) during the lockdown		< 0.001		< 0.001
Yes (*n* = 773)	43.3		35.3	
No (*n* = 963)	8.0		3.2	
Depression (mild to severe) during the lockdown		< 0.001		< 0.001
Yes (*n* = 776)	42.6		33.5	
No (*n* = 960)	10.9		4.5	
Anxiety (mild to severe) at follow-up		< 0.001		< 0.001
Yes (*n* = 745)	47.6		36.8	
No (*n* = 991)	6.7		2.9	
Depression (mild to severe) at follow-up		< 0.001		< 0.001
Yes (*n* = 209)	47.0		37.5	
No (*n* = 813)	8.1		3.5	
Suicidal thoughts at follow-up		< 0.001		< 0.001
Yes (*n* = 231)	67.0		56.6	
No (*n* = 1505)	21.2		11.5	
Sleep problems at follow-up		< 0.001		< 0.001
No (*n* = 654)	15.6		6.3	
A few (*n* = 794)	27.4		17.7	
A lot (*n* = 288)	52.8		42.2	
Perception of the traumatic events		< 0.001	–	
Acute stress (*n* = 632)	22.3		–	
Persistent stress (*n* = 414)	41.5		–	

^a^ Population: Respondents to the COCONEL May and June surveys, *N* = 1736

^b^ Combination of information collected in the first and the second survey

PTSD incidence was also higher in those diagnosed with COVID-19 (50.1%) than in those who were not (16.6%) (Table 2). Similarly, incidence was higher in people very concerned about developing this infection than in those who were not and increased significantly with media use. Finally, people who lived in an area strongly affected by COVID-19 had PTSD more often than the others.

PTSD incidence was significantly higher in participants with anxiety or depression, identified in either survey (Table 2). It was also significantly positively associated with sleep problems and suicidal thoughts at follow-up; only 7.0% of people without PSTD reported suicidal thoughts during the previous 2 weeks versus 43.1% of those with PTSD (*p* < 0.001). Finally, among people who had experienced a traumatic event, PTSD incidence was significantly higher in respondents reporting persistent stress due to this event than in those reporting acute stress only.

### Factors associated with PTSD incidence: multiple regressions

The first multiple regression model (Model 1, Table 3) showed that high media use during the lockdown, at least mild anxiety during the lockdown, and COVID-19 infection were significantly associated with an increased risk of PTSD 1 month later. Conversely, low media use (< 1 hr./day) during lockdown was negatively associated with PTSD. Sleep problems, at least mild symptoms of depression or GAD at follow-up were also positively associated with PTSD.

**Table 3:** Factors Associated with PTSD in the General Population (Model 1) and among the Population Exposed to a Traumatic Event (Model 2): Multiple Modified Poisson Regression Models (COCONEL 2020, *N* = 1736)

Characteristics of the respondents	Model 1. PTSD among the general population			Model 2. PTSD among the exposed population		
	RR	Adjusted RR	95% CI	RR	Adjusted RR	95% CI
**Gender**^**a**^ (ref: Women)						
Men	0.85	1.06	0.85–1.32	1.03	1.04	0.85–1.27
**Age**^**a**^ (ref: 35–64)						
< 35	1.43*	1.24	0.99–1.55	1.31*	**1.22**	1.00–1.48
> 64	0.85	0.96	0.70–1.33	0.91	1.05	0.77–1.43
**Media consumption of COVID-19-related information** (ref: 1- < 4 hrs)						
Low (< 1 hr)	0.53**	**0.67**	0.47–0.95	0.58**	**0.67**	0.49–0.93
High (= 4 hrs)	2.31***	**1.53**	1.18–1.97	1.88***	**1.40**	1.11–1.78
**Anxiety (mild to severe) during the lockdown** (ref: No)						
Yes	11.20**	**3.26**	1.95–5.46	1.62	**1.93**	1.22–3.06
**Diagnosed with COVID-19** (ref: No)						
Yes	3.01***	**1.43**	1.13–1.80	2.15***	1.16	0.91–1.47
**Anxiety (mild to severe) at follow-up** (ref: No)						
Yes	12.61***	**3.02**	1.77– 5.17	7.13***	**2.62**	1.61–4.27
**Depression (mild to severe) at follow-up** (ref: No)						
Yes	10.74***	**2.44**	1.53–3.90	5.79***	**1.94**	1.27–2.97
**Sleep problems at follow-up** (ref: Yes, a few/No)						
Yes, a lot	3.35***	**1.51**	1.22–1.88	2.26***	**1.36**	1.12–1.65
**Persistent perceived stress** (ref: No)						
Yes	NI^c^		–	1.86***	1.13	0.93–1.38
**Types of traumatic event** (ref: Other^d^)						
Work situation	NI		–	2.12	**2.19**	1.14–4.21
Government announcements	NI		–	1.45	1.31	0.65–2.62
Travel	NI		–	1.86	**2.17**	1.08–4.36
Several of the items proposed	NI		–	3.62***	**2.40**	1.36–4.25

^a^ Population: Respondents to the COCONEL May and June surveys, N = 1736

^b^ Covariates included in the model were selected in an automatic selection process, Stepwise option (p threshold: 5%)

^c^ NI: Covariate not included in the model

^d^ Other included: conflict with a relative, relative infected by the coronavirus, death of a relative and other not classified

*p < 0.5; ** p < 0.1; ***p < 0.001

Among people reporting traumatic events during the lockdown, Model 2 found a significantly higher risk of PTSD among respondents younger than 35 years, and for those with multiple traumatic events, or a work-related traumatic event, or traveling.

## Discussion

This study aimed to investigate the incidence of PTSD in the French population 1 month after the end of the lockdown period. Six respondents in ten reported traumatic experiences during this period, and most of them reported several types of events. Among those with at least one traumatic event, 30.1% had PTSD 1 month after the lockdown ended, while the incidence was 17.5% (95% CI = 15.7–19.3) in the general population. The risk of PTSD in the general population was higher in people with high COVID-19-related media use, with at least mild anxiety during the lockdown, and diagnosed with COVID-19. PTSD was also strongly comorbid with anxiety, depression, and sleep problems at follow-up. Among people reporting a traumatic event, young age and exposure to multiple types of traumatic events were associated with increased PTSD risk.

Several studies have measured PTSD incidence during this pandemic, with various instruments and definitions. Some measured acute stress, e.g., at the epidemic peak [5–8, 11, 22]. Others measured PTSD in specific groups: people aged 18-30 [22], or hospitalized for COVID-19 [9], or healthcare workers [7, 13]. Some of these samples showed marked selection [6, 9, 12, 13]. These methodological variations make comparisons between studies difficult, especially given the different cultural contexts. These studies report PTSD incidence after the lockdown ranging from 4.6 to 31.8%. Our definition of PTSD followed the American Psychiatric Association (APA) guidelines rather than using the more common global score approach. This choice should have improved our screening, because we used each PTSD symptom cluster to identify PTSD, taking their different specificity values into account [23].

Most recent review study have highlighted that health professional was concerned but also COVID-19 patients and general population [24]. Studies have found higher PTSD risks in women than men [6–12]; this gender imbalance has also been observed for the lockdown’s impact on anxiety, depression, and sleep problems [14, 15]. This association did not appear in this study of PTSD incidence, most likely because our inclusion of anxiety and depressive disorders in our multivariate analyses captured at least part of the gender effect (Table 3). Our finding that young people exposed to traumatic events during the lockdown are at higher risks of PTSD is consistent with previous findings [10, 22, 23]. Young people, especially those with precarious jobs, may suffer more than other population segments from the pandemic’s direct economic consequences, as during earlier health crises [24, 25]. The health risks might have compromised their education, and possibly their entry into the labor market. They may also be more highly exposed to stressful information than the rest of the general population [26] and more vulnerable to aspects of the lockdown, including isolation, social distancing, the closing of places young people gather to socialize, and reduced outdoor activities. All these factors could have made them more vulnerable to traumatic events.

As in recent studies [5, 9, 12, 15, 25], PTSD risk was higher in participants with direct exposure to COVID-19, especially in those diagnosed with it, and remarkably in people indirectly exposed via high media use. Conversely, low media exposure was associated with a low PTSD risk. This result adds to previous observations of a positive relation between media exposure to information about this illness and psychological distress, anxiety, and depression [15, 26]. Ahern et al. [27] observed a similar relation between PTSD occurrence and media use after the World Trade Center attacks of September 11, 2001, although the nature and intensity of the trauma (e.g., viewing defenestration) differed greatly from what the media showed during the COVID-19 health crisis (e.g., patients in intensive care and daily recitals of the number of new and total deaths). Our findings support the recommendation by Olagoke et al. [26] that public health professionals should work with the media to provide more content about mental health resources in pandemic situations, especially during lockdowns when people are more highly exposed to media coverage than usual. Moreover, our results suggest that probable GAD during the lockdown was predictive of PTSD a month later; evidence indicating that individuals with a history of psychological disorders are at higher risk of PTSD is now supported by several post-COVID-lockdown studies [10, 11]. The media could participate in prevention programs to encourage people with anxiety symptoms during a lockdown to seek care. Trained health professionals could thus provide individual PTSD prevention care.

The collection of data in two waves a month apart allowed us to explore the persistence of the perception of stress related to traumatic events. While acute stress was perceived more frequently than persistent stress, the latter was more highly correlated to incident PTSD, although no longer significantly after comorbidity factors were included in the model. PTSD–depression comorbidity has frequently been noted in the literature, among military personnel (exposed to combat), victims of sexual assault [30], and even students during the COVID-19 epidemic [6]. Previous population-based studies have also highlighted PTSD–anxiety comorbidity [28]. Similarly, our results about sleep disorders are consistent with earlier findings of serious sleep problems in PTSD patients [10, 29], including in a recent Chinese study during its lockdown [8]. As with comorbid depression, the causal relation between sleep disorders and PTSD is complex and partly reciprocal. Although nightmares of the traumatic event are included in the DSM-5 diagnostic criteria for PTSD, sleep disorders may be both a risk factor and an outcome of PTSD [10, 30]. Doctors should be aware that people with anxiety and/or depression symptoms and/or sleep problems, even some time after lockdown, may also suffer from PTSD and should thus routinely screen for it. Clinicians could systematically use tools -e.g. the Short PTSD Rating Interview (SPRINT)- to investigate PTSD in people with these symptoms. Moreover, the strong association we observed between PTSD and suicidal thoughts underlines the potential suicidal risk in patients with PTSD. Previous findings have shown that this risk is non-negligible in people with PTSD [20], especially those with psychiatric comorbidity. Assessing the suicide risk in caring for patients with PTSD after COVID-19 lockdowns is essential.

More generally, our findings raise the question of the nature of traumatic events in a pandemic containment context. Risk was highest for those reporting several sources of traumatic events. Government announcements alone were not associated with PTSD risk in the multivariate analysis, but were rather mostly cited by people exposed to several types of events during the lockdown. Therefore, in addition to other stressful events, these announcements may have played a role in augmenting the risk. The DSM-5 definition of trauma, however, requires “actual or threatened death, serious injury, or sexual violence”: proximity to death and/or physical violence was infrequent in the traumatic events reported in our study and is reflected in the low prevalence of intrusion symptoms in our results (Additional file 1: Table 1). Nonetheless, the DSM-5 definition has been controversial [31, 32], with several studies reporting that events classically considered nontraumatic (such as losing one’s job) are nevertheless associated with higher rates of PTSD than events considered traumatic [32]. Our findings suggest that the combination of several perceived traumatic events — even if they rarely involved proximity to death or violence — could expose people at high risk to PTSD in situations such as a pandemic-related lockdown. North et al. [23] suggested a new PTSD-like syndrome, resulting from a “nontraumatic” stressor, could be named “post-stressor stress disorder”. This expression might be applied to our findings as we await further studies to clarify the traumatic nature of each reported event during COVID-19-related lockdowns. Nevertheless, the daily presentation of the death toll in the media might suffice to meet the criterion of proximity to death required by DSM-5, and forced isolation, deprivation of liberty, the loss of a job and income could be viewed as social violence. Further research, particularly clinical, is needed to confirm these hypotheses and to explore in more detail the nature of the trauma experienced by people with PTSD a month after the COVID-19-related nationwide lockdown. Follow-up of these people for months will be key.

This study has some limitations. First, because the lockdown obviously affected data collection activities, online surveys were used to administer questionnaires. While effective, online surveys may involve some bias, in view of the risk that segments of the general population might be missed. The risk is nonetheless limited, given that 89% of French households have internet access, according to a 2018 estimate [33]. Moreover, the invitation email did not mention the study topic, which may have limited potential selection bias due to non-observed factors. While the PCL-5 used to define PTSD is a well-established and often used instrument, it is not a diagnostic instrument; the lack of clinical assessment is a limit of our population-based study. Psychiatric examinations are necessary in patients whose PTSD has been detected by the PCL-5. This should be organized in cohort studies to follow patients for sufficient time to study the course of PTSD, detect cases occurring sometime after the lockdown, and evaluate the impact of the second wave of COVID-19.

In conclusion, this study is the first to document with a longitudinal design the incidence of PTSD in the French population, a month after the first COVID-19 lockdown ended. It contributes to the demonstration of the psychological impact of the pandemic in the French population and suggests the need for more psychological support and a nationwide mental health promotion program in the general population and in specific groups. PTSD prevention and treatment should focus especially on young people with a history of anxiety, those who spend substantial time following COVID-19 in the media, and those with comorbidities. Recommendations should be developed for GPs about the detection of PTSD and how to deal with probable cases.

## Supplementary Information

**Additional file 1: Figure 1.** Time that the event was reported, during the lockdown (first survey) or/and one month later (second survey), and incidence of PTSD. **Table 1.** PTSD score according to population characteristics among those who experienced a traumatic event during the lockdown (COCONEL 2020, *N* = 1046).

## Data Availability

Data cannot be shared publicly because they are confidential; they include sensitive data such as health information. Data are available from the Aix Marseille Université, IRD, AP-HM, SSA, VITROME Institutional Data Access after agreement from the University Hospital Méditerranée Infection Ethic Commitee (contact: IHU – Méditerranée Infection 19-21 Boulevard Jean Moulin 13005 Marseille, or pierre-edouard.fournier@univ-amu.fr, with the number #2020-018) for researchers who meet the criteria for access to confidential data.

## Abbreviations

PTSD: Post-traumatic Stress Disorder
GAD: Generalized Anxiety Disorder
EHI: Equivalized Household Income
