# Supplementary material for: Incidence of PTSD in the French population a month after the COVID-19 pandemic-related lockdown: evidence from a national longitudinal survey
Source: BMC Public Health. 2022 Aug 5;22:1500. doi: 10.1186/s12889-022-13880-9 (PMC9356417; doi:10.1186/s12889-022-13880-9)
Supplement: Supplementary file 1 — Additional file 1: Figure 1. Time that the event was reported, during the lockdown (first survey) or/and one month later (second survey), and incidence of PTSD. Table 1. PTSD score according to population characteristics among those who experienced a traumatic event during the lockdown (COCONEL 2020, N = 1046). [file 12889_2022_13880_MOESM1_ESM.docx]

**SUPPLEMENTAL MATERIAL**

Figure 1. Time that the event was reported, during the lockdown (first survey) or/and one month later (second survey), and incidence of PTSD.

*Population: Respondents to the COCONEL May and June surveys (N=1,736).*

Table 1. PTSD score according to population characteristics among those who experienced a traumatic event during the lockdown (COCONEL 2020, N=1,046).

|  | **PTSD**  **score** | **Subscore A: Intrusion** | **Subscore B: Avoidance** | **Subscore C: Negative alteration in cognition and mood** | **Subscore D: Increased arousal** |
| --- | --- | --- | --- | --- | --- |
| **All** | 21.1 | 4.8 | 2.1 | 7.5 | 6.7 |
| *Sociodemographic characteristics* | | | | | |
| **Gender** | ns | ns | ns | ns | Ns |
| Men | 20.1 | 4.5 | 2.0 | 7.3 | 6.5 |
| Women | 21.7 | 5.0 | 2.2 | 7.7 | 6.8 |
| **Age** | * | * | ns | * | Ns |
| < 35 years | 23.5 | 5.4 | 2.2 | 8.6 | 7.3 |
| 35 - 64 years | 20.7 | 4.7 | 2.1 | 7.2 | 6.7 |
| > 64 years | 18.7 | 4.1 | 2.0 | 6.8 | 5.8 |
| **Education Level** | ns | ns | ns | ns | ns |
| No high school diploma | 22.5 | 5.3 | 2.3 | 8.0 | 6.9 |
| High school diploma | 20.2 | 4.4 | 2.0 | 7.2 | 6.6 |
| Undergraduate degree | 20.3 | 4.5 | 2.0 | 7.3 | 6.6 |
| Postgraduate degree | 18.8 | 4.1 | 1.7 | 6.9 | 6.1 |
| **EHI** | ** | * | ns | * | * |
| Low (q1) | 25.2 | 5.8 | 2.6 | 8.9 | 7.9 |
| Middle (q2-q3) | 19.6 | 4.3 | 2.0 | 7.1 | 6.2 |
| High (q4) | 18.7 | 4.2 | 1.7 | 6.7 | 6.1 |
| Missing | 23.9 | 5.8 | 2.2 | 8.3 | 7.5 |
| **Parent of young children (<14 years)** | ns | ns | ns | ns | ns |
| Yes | 19.9 | 4.6 | 1.9 | 7.0 | 6.3 |
| No | 21.4 | 4.9 | 2.1 | 7.7 | 6.8 |
| *Health condition reported during the lockdown* | | | | | |
| **Chronic health problem or chronic disease** | *** | *** | *** | ** | *** |
| Yes | 24.1 | 5.6 | 2.5 | 8.4 | 7.6 |
| No, don't know | 19.7 | 4.4 | 1.9 | 7.1 | 6.2 |
| **Consulted for psychological issues in the 12 months before lockdown** | *** | *** | *** | *** | *** |
| Yes | 30.9 | 7.2 | 3.1 | 11.1 | 9.4 |
| No | 19.2 | 4.3 | 1.9 | 6.8 | 6.1 |
| *COVID-19 exposure* | | | | | |
| **Living in an area strongly affected by COVID-19** | ** | ** | * | ** | ** |
| Yes | 23.0 | 5.3 | 2.3 | 8.2 | 7.3 |
| No | 19.6 | 4.4 | 1.9 | 7.0 | 6.2 |
| **Media consumption per day of information about COVID-19** | *** | *** | *** | *** | *** |
| Low (<1 hr) | 15.5 | 3.2 | 1.4 | 5.7 | 5.1 |
| Intermediate (1- <4 hrs) | 19.0 | 4.3 | 1.9 | 6.6 | 6.2 |
| High (≥4 hrs) | 29.5 | 7.1 | 3.0 | 10.5 | 8.9 |
| **Diagnosed with COVID-19** | *** | *** | * | *** | *** |
| Yes | 32.8 | 8.2 | 2.8 | 11.6 | 10.2 |
| No | 20.6 | 4.7 | 2.1 | 7.4 | 6.5 |
| **Serious concern about being infected by the coronavirus** | *** | *** | *** | *** | *** |
| Yes | 31.3 | 7.7 | 3.3 | 10.6 | 9.7 |
| No | 19.5 | 4.4 | 1.9 | 7.0 | 6.2 |
| *Mental health symptoms* | | | | | |
| **Anxiety (mild to severe) during the lockdown** | *** | *** | *** | *** | *** |
| Yes | 27.7 | 6.5 | 2.7 | 9.8 | 8.7 |
| No | 10.0 | 2.0 | 1.0 | 3.7 | 3.3 |
| **Depression (mild to severe) during the lockdown** | *** | *** | *** | *** | *** |
| Yes | 27.8 | 6.4 | 2.7 | 9.9 | 8.8 |
| No | 10.8 | 2.3 | 1.1 | 3.9 | 3.4 |
| **Anxiety (mild to severe) at follow-up** | *** | *** | *** | *** | *** |
| Yes | 29.6 | 6.9 | 2.9 | 10.4 | 9.3 |
| No | 9.7 | 1.9 | 0.9 | 3.6 | 3.2 |
| **Depression (mild to severe) at follow-up** | *** | *** | *** | *** | *** |
| Yes | 29.4 | 6.8 | 2.9 | 10.4 | 9.3 |
| No | 10.2 | 2.2 | 1.1 | 3.7 | 3.3 |
| **Suicidal thoughts in the past two weeks** | *** | *** | *** | *** | *** |
| Yes | 35.5 | 8.6 | 3.5 | 12.7 | 10.8 |
| No | 17.6 | 3.9 | 1.8 | 6.3 | 5.7 |
| **Sleep problems at follow-up** | *** | *** | *** | *** | *** |
| No /Yes, a few | 17.7 | 4.0 | 1.7 | 6.5 | 5.5 |
| Yes, a lot | 32.3 | 7.6 | 3.2 | 11.0 | 10.5 |
| **Types of traumatic event** |  |  |  |  |  |
| Work situation | 17.7 | 4.0 | 1.7 | 6.5 | 5.5 |
| Government announcements | 16.2 | 3.8 | 1.6 | 5.6 | 5.1 |
| Travel | 15.0 | 2.6 | 1.4 | 5.7 | 5.4 |
| Several | 25.2 | 5.8 | 2.5 | 9.0 | 7.9 |
| Other (conflict with a relative, relative infected by the coronavirus, death of a relative and other not classified) | 12.9 | 2.7 | 1.1 | 4.5 | 4.6 |

*Population: Respondents to the COCONEL May and June surveys who reported a traumatic event during the lockdown (N=1,046).*
